# Solubilized β-Glucan Supplementation in C57BL/6J Mice Dams Augments Neurodevelopment and Cognition in the Offspring Driven by Gut Microbiome Remodeling

**DOI:** 10.3390/foods13193102

**Published:** 2024-09-28

**Authors:** Dorsilla A. Katimbwa, Yoonsu Kim, Min Jeong Kim, Minsoo Jeong, Jinkyu Lim

**Affiliations:** 1 Department of Food Biomaterials, Kyungpook National University, Daegu 41566, Republic of Korea; dorsillaanono@knu.ac.kr; 2 Department of Integrative Biology, Kyungpook National University, Daegu 41566, Republic of Korea; 3 School of Food Science and Biotechnology, Kyungpook National University, Daegu 41566, Republic of Korea; 4 Department of Applied Biosciences, Kyungpook National University, Daegu 41566, Republic of Korea

**Keywords:** oat β-glucan, neurodevelopment, perinatal and gestational gut microbiome profile, neurodevelopment, later-life cognition

## Abstract

A maternal diet rich in dietary fiber, such as β-glucan, plays a crucial role in the offspring’s acquisition of gut microbiota and the subsequent shaping of its microbiome profile and metabolome. This in turn has been shown to aid in neurodevelopmental processes, including early microglial maturation and immunomodulation via metabolites like short chain fatty acids (SCFAs). This study aimed to investigate the effects of oat β-glucan supplementation, solubilized by citric acid hydrolysis, from gestation to adulthood. Female C57BL/6J mice were orally supplemented with soluble oat β-glucan (ObG) or carboxymethyl cellulose (CMC) via drinking water at 200 mg/kg body weight during breeding while the control group received 50 mg/kg body weight of carboxymethyl cellulose. ObG supplementation increased butyrate production in the guts of both dams and 4-week-old pups, attributing to alterations in the gut microbiota profile. One-week-old pups from the ObG group showed increased neurodevelopmental markers similar to four-week-old pups that also exhibited alterations in serum markers of metabolism and anti-inflammatory cytokines. Notably, at 8 weeks, ObG-supplemented pups exhibited the highest levels of spatial memory and cognition compared to the control and CMC groups. These findings suggest a potential enhancement of neonatal neurodevelopment via shaping of early-life gut microbiome profile, and the subsequent increased later-life cognitive function.

## 1. Introduction

The effects of perinatal malnutrition on metabolic maladaptation and disease risk in offspring are central to the “fetal origins of adult disease” hypothesis [1,2]. Dietary adaptations during gestation and breastfeeding play a significant role in shaping the gut microbiome of offspring, particularly in the establishment of core microbiota during this period [3,4,5,6]. The resulting changes are essential as energy demands increase significantly during the perinatal period [7].

The core microbiota established in the gut influences the body’s response to dietary changes [8]. There are significant interindividual variations in the relative abundance of core taxa depending on factors such as region, diet, and disease [9]. The ability of various microbes to utilize available energy sources is a key determinant of their capacity to colonize, thrive, and proliferate in the gut [10]. On average, the *Bacteroidetes* genome contains more carbohydrate-active digestive enzymes compared to Firmicutes. However, within the *Firmicutes* phylum, bacteria such as *Clostridium hathewayi*, *Clostridium bolteae*, and *Ruminococcus bromii* represent the largest proportion of genes related to fiber digestion [10,11,12]. Conversely, members of *Bacteroidetes* encode hydrolases that are secreted into the intestinal lumen, playing a crucial role in fiber digestion [10]. Studies suggest that although *Bacteroidetes* are the primary degraders of many complex carbohydrates, the ratio of *Bacteroidetes* to *Firmicutes* is a key factor in determining the overall capacity for carbohydrate digestion [13,14].

The critical periods for maternal influence on the immune education of offspring occur during gestation, immediately after birth, and throughout lactation [15,16]. Maternal–fetal immune crosstalk at the placental interface remains an active area of research [17,18,19]. One study found that a high-fiber diet resulted in increased propionate levels in the gut of pregnant mice, lowering the offspring’s predisposition to obesity by suppressing insulin signaling and downregulating fat deposition in adipocytes [20]. Maternal diet-induced changes in the growth of specific bacterial species, alongside similarities in IgA expression in the gut and milk of dams, suggest a potential pathway for matrilineal transmission of immunological factors [21]. Several studies have demonstrated that antenatal exposure to short-chain fatty acids (SCFAs), such as acetate, propionate, butyrate, iso-butyrate, valerate, and iso-valerate, may exert immune-modulating effects that protect against conditions such as asthma in offspring [22]. Placental and intrauterine inflammation associated with maternal obesity has been shown to alter fetal cytokine expression, leading to neuronal and systemic inflammation [23,24].

β-glucans are dietary fibers composed of polymeric D-glucose monomers containing β-1,3 linkages mixed with β-1,4 or β-1,6 linkages depending on their source [25,26,27]. These fibers are found in various sources, including mushrooms, seaweeds, yeast, cereals, and certain bacteria [28]. Among cereal-derived β-glucans, those extracted from wheat, millet, triticale, and sorghum have been extensively researched, with a majority of studies focusing on barley, oats, rye, and rice [29,30,31,32,33,34]. Cereal-derived β-glucans primarily consist of β-1,3 and β-1,4 linkages and are predominantly located in the aleurone, sub-aleurone layers, and kernel [35,36].

Numerous studies have demonstrated the potential of β-glucans from various sources to enhance neurodevelopment and cognitive function [37]. In one study, the effects of dietary fiber supplementation in mice using β-glucans extracted from mushrooms, insoluble oat bran, and curdlan were compared. The findings indicated a similarity in the regulation of the microbiota–gut–brain axis across these different fiber sources [38]. The study demonstrated promising benefits for both memory and overall health highlighting the role of the *Muribaculaceae* family (formerly known as Bacteroidales_S24-7 family). These improvements were associated with elevated levels of brain-derived neurotrophic factor (BDNF) and post-synaptic density protein 95 (PSD95) in the prefrontal cortex, a critical region of the brain for memory function. This upregulation may be attributed to extracellular signal-regulated kinase 1/2 (ERK1/2)-mediated BDNF synthesis [39]. Additionally, these β-glucans have been reported to modulate the expression of immune factors, including increased IL-10 and reduced IL-6 and TNF-α, potentially promoting a more balanced immune response in the brain [38]. This immunomodulatory effect has also been observed in mice fed a high-fat diet [40,41]. Notably, oat bran β-glucan uniquely altered gut microbiota composition and enhanced intestinal mucus production, highlighting potential gut-specific benefits [38].

The gut bacterial consortium is essential for maintaining immune system homeostasis in mammals [42]. Several studies have demonstrated the anti-inflammatory effects of SCFAs, such as butyrate and propionate when absorbed by the body [43,44]. These effects result from the production of SCFAs as primary metabolites of fiber digestion [13,14]. Maternal gut microbiota dysbiosis has been implicated in the development of neurological disorders in offspring through microbially modulated metabolites, alterations in the composition of the offspring’s microbiota, or by mediating immune activation and increasing IL-17a levels [45,46,47,48,49,50,51]. Mild to moderate food restriction (30%) in pregnant mice has been shown to trigger a premature leptin surge, disrupting energy regulation in the hypothalamus and resulting in adverse metabolic sequelae in the later stages of the offspring’s life [52]. Overexpression of TNF-α has been shown to impair hippocampal function [53], whereas IL-6 has been demonstrated to suppress long-term potentiation (LTP) [54].

The primary objective of this study was to develop a non-toxic, cost-effective solubilization process to achieve high yields of high-purity soluble β-glucan from oats, barley, sorghum, and millet. Malic and citric acid treatments, in conjunction with various temperatures, were tested for increasing durations. The solubilized oat β-glucan with the highest yield and purity was selected to investigate neurodevelopmental differences in 1-week and 4-week-old offspring of mice. These mice were fed either solubilized oat β-glucan or soluble carboxymethyl cellulose (CMC), with low soluble CMC intake, functioning as the control. Furthermore, the long-term effects of these treatments were evaluated through cognitive and behavioral assessments in pups, from the eighth to the eleventh week. The impact of solubilized oat β-glucan supplementation on gut microbiota composition and SCFA production was analyzed in pregnant female C57BL/J mice and their offspring at 4 weeks of age (weaning). Changes in circulating cytokine levels were also measured in the weaning offspring.

## 2. Materials and Methods

### 2.1. Production of Purified β-Glucans from Oat Products

#### Extraction, Solubilization, and Purification of β-Glucans

The procedure for the solubilization and purification of β-glucan from cereals was adopted from various protocols and optimized for citric and malic acids [55,56,57,58]. To optimize the methodology for β-glucan solubilization, high β-glucan content oat-based products were selected. These included OatWell^®^ oat bran β-glucan powder, containing 3 g of β-glucan per 14 g serving (abbreviated as HBOB; Henry Bloom, NSW, Australia), and oat bran with 28% β-glucan content (abbreviated as AOB; Woolworths, NSW, Australia). Additionally, β-glucans were extracted from raw cereals, including barley, oats, sorghum, and millet. These cereals were first defatted by creating a 30% *w*/*v* flour suspension in hexane, followed by stirring for 2 h at room temperature (23.9–25 °C RT).

For solubilization, 10 g of HBOB and AOB were each mixed with 100 mL of 0.1 M citric acid and malic acid. Distilled water was used as the control. The slurry was then heated in a water bath at 85 °C for either 8 or 16 h. Subsequently, the slurry was mixed with 100 mL of distilled water, stirred, and centrifuged at 10,000× *g* for 20 min at RT. The resulting supernatant was freeze-dried for 48 h and re-dissolved in a minimal amount of distilled water. Samples were taken from each condition, and the yield was calculated.

For purification, 100 g of HBOB and AOB was mixed with 200 mL of 0.1 M sodium phosphate buffer (pH 6) and then combined with 20 units of α-amylase, pre-warmed to 40 °C in a water bath. The mixture was incubated for 12 h at 40 °C with gentle stirring. This process resulted in a thick slurry that retrograded to form a solid lump and a yellow aqueous solution which was discarded. The solid lump was then blended with 500 mL of 0.1 M citric acid, and an additional 200 mL of citric acid solution was added to the slurry. The mixture was incubated for 16 h at 85 °C. After incubation, the slurry was centrifuged at 10,000× *g* for 20 min at RT. The separated β-glucan-rich viscous supernatant was retained for further purification. The pH of the supernatant was adjusted to 7.4 using 1 M sodium hydroxide, after which 40 mg of pancreatin (from porcine pancreas, Sigma-Aldrich, St. Louis, MO, USA) dissolved in 10 mL of 0.1 M sodium phosphate buffer (pH 6) was added. The mixture was then incubated at 40 °C for 12 h with gentle stirring. The resulting solution was freeze-dried and subsequently redissolved in 400 mL of distilled water. To reduce viscosity, the viscous solution was heated at 80 °C for 30 min before being transferred into a dialysis tubing with a molecular weight cut-off of 6000–8000 Da (Spectrum^®^ Laboratories Inc., Compton, CA, USA). The dialysis bags were placed in a larger container containing distilled water at ten times the volume of the sample per bag and gently stirred overnight. The water in the container was replaced every 3 h for three additional times. The final samples were then assayed for β-glucan content.

### 2.2. Quantification of β-Glucan

#### 2.2.1. Macromolecular Characteristics of Solubilized Oat β-Glucan

The β-glucan content of the extracts was analyzed using the mixed-linkage β-glucan assay kit (Megazyme International Ltd., Bray, Co., Wicklow, Ireland), with minor modifications to account for the expected high concentration of β-glucan in the cereal extracts [59,60]. The initial incubation step at 100 °C was extended to 30 min. The amount of lichenase used in the subsequent step was doubled, and the mixture was incubated at 50 °C for 2 h. The reaction solution was then mixed with 50 µL of sodium acetate buffer (200 mM, pH 4) and stirred for 15 min. The remainder of the procedure followed the protocol outlined in the kit. β-glucan content was calculated using Megazyme Mega-Calc™ (Megazyme International Ltd., Bray, Co., Wicklow, Ireland) and presented as an average percentage ± standard error of the mean (SEM).

#### 2.2.2. Monosaccharide Analysis

A 20 mg sample of solubilized β-glucan was mixed with 2 M trifluoracetic acid (TFA) and hydrolyzed at 110 °C for 12 h. The resulting hydrolysate was combined with methanol and evaporated in vacuo to remove the TFA, and then reconstituted in 2 mL of high-performance liquid chromatography (HPLC)-grade water. Before injection, the hydrolysate was filtered through a polyvinylidene fluoride (PVDF) filter membrane (0.45 µm), and the sugars were detected and quantified using HPLC (Agilent 1260 HPLC, Santa Clara, CA, USA) [61].

#### 2.2.3. Molecular Weight Analysis

The molecular weight (Mw) and polydispersity index (Mw/Mn) of solubilized β-glucans were measured using gel permeation chromatography (GPC) (Waters, Milford, MA, USA), coupled with a refractive index (RI) detector (Waters 2414, Milford, MA, USA) utilizing ultra-hydrogel columns (120, 250, 500, and Linear) [61]. Solubilized β-glucan samples (1%, *w*/*v*) were dissolved in 20 mM NaNO_3_, filtered (0.45 µm), and injected into the GPC system. The elution was performed using 0.02 M NaNO_3_ at 30 °C with a flow rate of 0.8 mL/min. The calibration curve of PEG standards (1000–40,000 gmol^−1^) was used for molecular weight determination.

### 2.3. Animal Experiment

#### 2.3.1. Animal Diet

**Figure 1:**
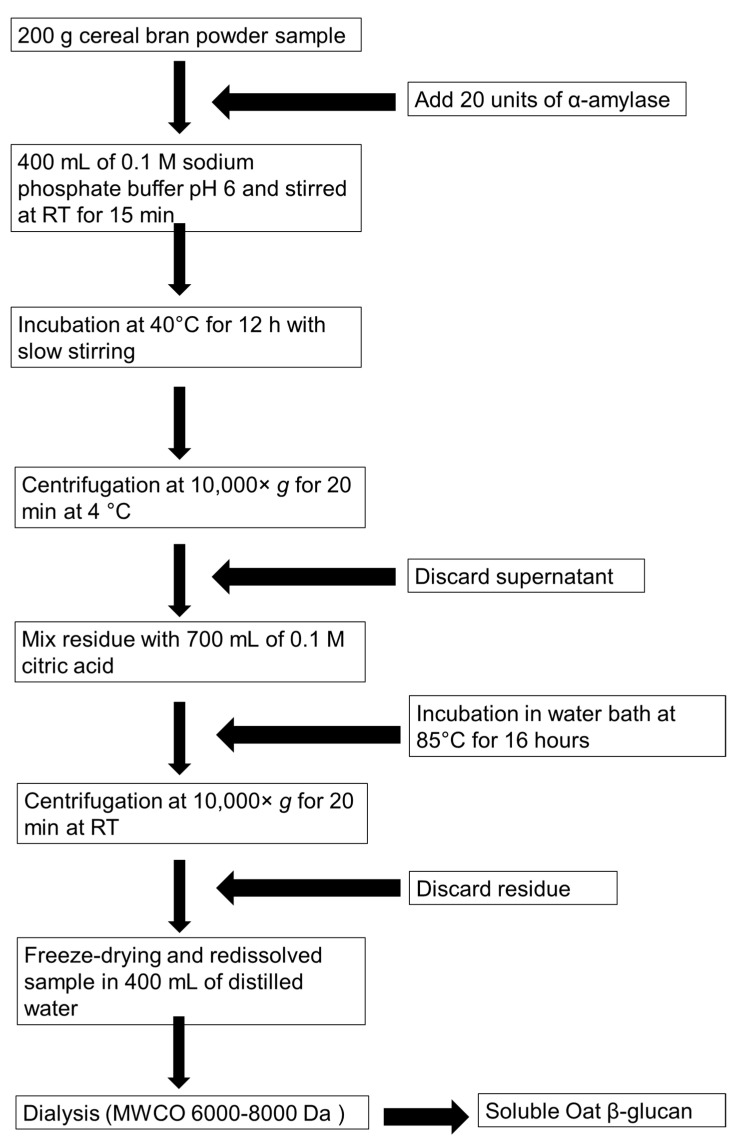
Procedure for solubilization and purification of β-glucans from cereals.

Solubilized oat β-glucan was prepared as described in Figure 1 using oat bran (abbreviated as AOB; Woolworths, NSW, Australia) as raw material. Soluble cellulose was procured in the form of carboxymethyl cellulose sodium salt (C5678, Sigma-Aldrich, St. Louis, MO, USA). The research diet was formulated based on the “AIN-93 Diet for Laboratory Rodents” formulated by Research Diet (New Brunswick, NJ, USA).

#### 2.3.2. Soluble Fiber Supplementation in Dams

To assess the effect of solubilized oat β-glucans on the offspring of female C57BL6/J mice, 6-week-old mice of similar body weight were acclimated for one week. All animal procedures were conducted in accordance with the guidelines of the Committee on the Care and Use of Laboratory Animals at Kyungpook National University (approval number: 2022-0392). Throughout the study, the mice were provided ad libitum access to a research diet with no fiber content. This zero-fiber diet was formulated by Research Diet (New Brunswick, NJ, USA) based on the “AIN-93 Diet for Laboratory Rodents”, in which microbiota-accessible carbohydrates (cellulose) were replaced with simple carbohydrates or monosaccharides (sucrose and corn starch). The diet was supplemented with solubilized oat β-glucans or carboxymethyl cellulose, both administered orally via drinking water. Three test groups were established: (1) the Control group, which received 50 mg/kg body weight (bw) of carboxymethyl cellulose, (2) the CMC group which received 200 mg/kg bw of carboxymethyl cellulose, and (3) the ObG group, which received 200 mg/kg bw of solubilized oat β-glucans. At 9 weeks of age, females from each group were bred with male mice maintained on the Control group diet. During gestation and lactation, the female mice continued their respective pre-breeding diets.

#### 2.3.3. Collection of Samples from Dams and Perinatal Pups

One and four weeks after birth, offspring (*n* = 8 per group) were sacrificed. The mice were fasted for 10 h before being euthanized by cervical dislocation or CO_2_ exposure, followed by immediate blood collection and brain excision. The small and large intestines were snap-frozen on dry ice and then stored at −80 °C for further analysis. Cecal matter was extracted and mixed with 1 mL phosphate-buffered saline (PBS) or directly snap-frozen on dry ice, then stored at −80 °C until further analysis. Cecal matter from the dams was collected similarly, one week after the births.

#### 2.3.4. Long-Term Supplementation of Offspring

At 4 weeks of age, the pups (*n* = 8) were separated from the dams and by gender and fed the same diets as their respective mothers. At 8 weeks, these pups were subjected to Y-maze, passive avoidance, and Morris water maze tests over a 3-week period. At 11 weeks, the offspring were euthanized as described in the animal diet section.

### 2.4. Cognitive and Behavioral Tests

#### 2.4.1. Y-Maze Test

The Y-maze test is used to evaluate working memory by measuring the spontaneous alternation behavior of mice [62] as they explore three plastic arms (35 cm long, 25 cm high, and 10 cm wide). Rodents typically explore a new arm of the maze rather than returning to one they have previously visited. The hippocampus and prefrontal cortex are involved in this task. Each mouse underwent a single 5-min test, during which it was placed in a designated arm of the maze, and the total number of distinct arm entries was recorded. Spontaneous alternations were defined as consecutive entries into three different arms.

#### 2.4.2. Passive Avoidance Test

The passive avoidance test is used to evaluate fear conditioning as well as learning and memory abilities [63]. The apparatus used for this test (Gemini Avoidance System, San Diego, CA, USA) consists of two chambers separated by a guillotine door. On the adaptation day, each experimental animal was placed in the light chamber for 10 s. The door was then raised, allowing the mice to enter the dark chamber. On the training day, when the mice moved from the light chamber to the dark chamber, the door was closed, and the mice received an electrical foot shock (5 mA) for 3 s through stainless-steel rods. On the test day, the mice were again placed in the light chamber for data acquisition, but no electrical foot shock was administered. The time taken for a mouse to move from the light chamber to the dark chamber after the door was opened was recorded as the latency time for both training and test trials.

#### 2.4.3. Morris Water Maze Test

The Morris water maze test is used to evaluate spatial learning and memory [64]. The test was conducted in a stainless-steel circular pool (90 cm in diameter and 45 cm in height) filled with water (22 ± 1 °C) to a depth of 30 cm. The pool was placed in a soundproof environment with dim lighting and divided into four zones. The mouse was placed in the pool, facing the wall, with a platform positioned at the center above the water’s surface. The time taken for the mouse to locate the platform was recorded, and the mouse was allowed to remain on the platform for an additional 3 s until the process was repeated three more times. Training was conducted for 4 d, and on day 5, the mice were tested. The mice were again placed at a designated spot on the pool’s wall, with the platform fully submerged approximately 1 cm below the surface of the stained water. The movement of the mice was recorded using video tracking software from a system positioned directly above the pool, and the time taken to reach the platform was recorded for each mouse.

### 2.5. Cecal SCFA Quantitative Analysis

Cecal matter from 4-week-old pups was mixed with 1 mL of PBS and centrifuged at 6000× *g*. From the resulting supernatant, 150 µL was used for SCFA and protein quantitative analysis. Cecal matter from dams was weighed, and 100 mg of the sample was used for SCFA quantitative analysis. The SCFA analysis was performed using an Agilent Headspace 7697A coupled with an Agilent GC 7890B and a flame ionization detector (FID). Samples were mixed with GC buffer and an internal standard and passed through an HP-Innowax capillary column (30 m × 250 μm × 0.25 μm) using nitrogen (carrier gas), hydrogen, and air. Calibration curves were generated using acetic, propionic, butyric, iso-butyric, valeric, and iso-valeric acids, mixed with the internal standard.

### 2.6. 16s RNA Microbiome Profiling

#### 2.6.1. Cecal Sample 16s rRNA Sequencing

The cecal contents from four pups from each group were used to extract and assess genomic DNA using a QiAamp DNA Stool Mini Kit (Qiagen, Stockach, Germany). The sequencing of the bacterial 16S rDNA V3-V4 region was performed on an Illumina MiSeq platform, followed by pipeline analysis for 16S-based microbial profiling.

#### 2.6.2. Pre-Processing of Raw 16s rRNA Amplicon Sequencing Data

Raw reads obtained from the sequencing platform were preprocessed using DADA2 [65] to translate these reads into taxonomic information. The quality of the raw reads was initially evaluated, followed by trimming, truncation, and removal of chimeras based on the quality profile. The trimmed reads were then clustered into operational taxonomic units (OTUs), at a 97% similarity threshold and further refined into high-resolution amplicon sequence variants (ASVs). ASVs, identified based on their unique biological sequences, facilitate meta-analysis across studies. The sequenced reads are assigned taxa using a naive Bayesian classifier method, with a training set of reference sequences and known taxonomy from the Silva reference database [66,67,68,69]. This method outputs taxonomic assignments.

#### 2.6.3. Community Profiling

Microbial community diversity profiles were evaluated using α-diversity analysis, utilizing Chao1 and Shannon indices, as well as β-diversity analysis and principal coordinate analysis (PCoA) using phyloseq package on R statistical software (v4.3.3; R Core team 2024) [70]. Significant differences were assessed using a *t*-test. The taxonomic data were normalized and rarefied prior to visualization.

### 2.7. RNA Sequencing

#### 2.7.1. RNA Extraction, Purification, and Integrity Testing Using Cortex Samples from 4-Week-Old Pups

Freshly excised cortex and whole brain samples were placed in 1 mL and 500 μL of RiboEx™ reagent (GeneAll Biotechnology Co., Ltd., Seoul, Republic of Korea) in 1 mL tubes to extract total RNA, following the manufacturer’s instructions. Briefly, the samples were preserved in dry ice before homogenization. The homogenate was centrifuged at 6000× *g* for 10 min at 4 °C. The supernatant was transferred to a fresh tube and stored at −80 °C for up to 1 year until further analysis.

Total RNA was isolated from the RiboEx™ extract by adding 200 μL of chloroform, thoroughly mixing, and allowing the mixture to stand for 3 min. The mixture was then centrifuged at 6000× *g* for 15 min at 4 °C. The aqueous phase was transferred to a fresh tube and combined with 500 μL of isopropyl alcohol per 1 mL of RiboEx™ used in the initial homogenization. The mixture was incubated at RT for 10 min and then centrifuged at 6000× *g* for 10 min at 4 °C. The resulting pellets were washed three times with 1 mL of 75% ethanol and then centrifuged at 3600× *g* for 5 min at 4 °C. The pellets were air-dried and then redissolved in 100 μL of RNase-free water, followed by incubation at 60 °C for 10 min. The isolated RNA was further purified using an RNeasy mini kit (Qiagen, Hilden, Germany) according to the manufacturer’s instructions.

#### 2.7.2. Bioinformatic Analysis of RNA-Seq Data

Quality control, read mapping, and alignment were performed using Catadapt (Mercator Research Center Ruhr, Germany) and STAR 2 (University of Stuttgart, Germany) software. Reads mapped to known genes were obtained using HTSeq-count software v0.11.1 (Stanford University, Stanford, CA, USA), which normalized the data and generated count data. Differentially expressed genes (DEGs) were analyzed using the R package DESeq2 v1.42.0, which considers the library size and sequencing depth for each gene. Genes with zero read counts in all samples were excluded from downstream analyses. Genes with a log10-fold change greater than 2 were retained for downstream analyses.

Enrichment analysis of the identified DEGs was performed to identify enriched pathways and gene ontology (GO) terms using Metascape [71]. Significantly perturbed networks were identified by mapping DEGs from each phenotype onto pre-built process networks (Gene Ontology and Wikipathways). Process networks were sorted by *p*-value, and the top ten statistically enriched networks were reported.

### 2.8. RNA Isolation from Samples of 1-Week-Old Pup Brains and 4-Week-Old Pup Intestines, and Quantitative Real-Time PCR

Total RNA was isolated from samples of 1-week-old pup brains and 4-week-old pup intestines (from the jejunum to the colon) using RiboEx™ and purified using RNeasy mini kit (Qiagen, Hilden, Germany), as described in Section 2.7.1. To quantify mRNA, cDNA was synthesized from 1 μg of total RNA in a final volume of 20 μL using the Maxima H Minus First Strand cDNA synthesis kit (K1681; Thermo Fisher Scientific, Waltham, MA, USA). Quantitative real-time PCR (qPCR) was then performed using TOPreal™ qPCR 2X pre-mix (SYBR Green with low ROX, Daejeon, Republic of Korea) on a Light Cycler^®^ 96 version 1.1 PCR Detection System (F. Hoffmann-La Roche AG, Basel, Switzerland) under standard conditions, following the manufacturer’s instructions. Results were analyzed using the 2^−ΔΔCt^ method, with GAPDH and β-actin as internal standards. The primer sequences are listed in Table 1.

### 2.9. Western Blotting

Western blotting was performed on the brain samples of 1-week-old offspring. Proteins from brain and intestinal samples were extracted using a cell lysis buffer containing RIPA buffer (150 mM NaCl, 50 mM Tris-HCl pH 8.0, 1% NP-40, 0.1% sodium deoxycholate and 0.1% SDS, adjusted with distilled water), along with cOmplete™ Mini, EDTA-free protease inhibitor cocktail (Sigma-Aldrich, St Louis, MO, USA) and PhosSTOP™ phosphatase inhibitor cocktail (Sigma-Aldrich, St Louis, MO, USA). Equal amounts of protein per sample (20–40 µg) were loaded onto 10% SDS-PAGE gels and transferred to polyvinylidene difluoride (PVDF) membranes using a Bio-Rad electrophoresis system (Hercules, CA, USA). The membranes were blocked with 1% BSA in TBS for 2 h and then incubated overnight at 4 °C with various primary antibodies: PSD95 (#3450, Cell Signaling Technology, Boston, MA, USA), C3 (sc-28294, Santa Cruz Biotechnology, Dallas, TX, USA), and β-actin (sc-47778, Santa Cruz Biotechnology, Dallas, TX, USA). Secondary antibodies included HRP-conjugated anti-mouse and HRP-conjugated anti-rabbit antibodies (Thermo Fisher Scientific, Waltham, MA, USA). The antibody-bound proteins were visualized using the SuperSignal™ West Femto PLUS Chemiluminescent Substrate Kit mixed with the SuperSignal™ West Pico PLUS Chemiluminescent Substrate Kit (1:3), and the images were captured using ImageQuant LAS 4000 mini (GE Healthcare Life Sciences, Little Chalfont, UK). Protein quantification was done by analyzing the intensities of protein bands using ImageJ software v1.5k [72] and normalized to a protein concentration of samples determined by Bradford’s assay [73].

### 2.10. BDNF Assay

The levels of BDNF in the cortex of 4-week-old and 11-week-old offspring, as well as in the brains of 1-week-old offspring, were measured using the BDNF DuoSet kit (R&D Systems; DY248). In brief, 96-well microplates were coated with 100 μL of diluted capture antibody per well and incubated overnight at RT. On the second day, the plates were thoroughly washed and blocked with reagent diluent (300 μL, 1 h at RT). Subsequently, 100 μL of cortex or brain protein extract, standards in reagent diluent, or appropriate diluent were added to the prepared wells and incubated for 2 h at RT. Following this, 100 μL of detection antibody, 100 μL of working dilution of streptavidin-HRP, 100 μL of substrate solution, and 50 μL of stop solution were sequentially added to each well. Finally, the optical density of each well was measured using a microplate reader set to 540 nm. The BDNF concentration was expressed in pictograms of BDNF per microgram of protein.

### 2.11. Cytokine Array on Serum

Serum aliquots (20 µL) from each group were pooled to create two replicates of 100 µL, which were used for the cytokine array analysis. The serum was analyzed using a Proteome Profiler Mouse XL cytokine array (R&D Systems, Cat # ARY028) according to the manufacturer’s instructions. Immunoblot images were captured and visualized using the ChemiDoc MP imaging system (Bio-Rad, Hercules, CA, USA). Spot densities were quantified using Quick Spots image analysis software v25.6.0.3 from R&D Systems^®^. Cytokine spot intensities were measured with consistent area sizes and subtracted from the background intensity of adjacent areas. Data are presented as the mean of t arrays per analyte ± SEM.

### 2.12. Fluorescence Immunohistochemistry

Frozen whole brains were thawed on ice and then fixed in 4% paraformaldehyde in 0.1 M PBS overnight at 4 °C. For cryoprotection, the brains were immersed in 15% sucrose in 0.1 M PBS overnight, followed by a second immersion in 30% sucrose overnight at 4 °C. The fixed brains were then placed in molds containing optimal cutting temperature compound (OCT) and covered with additional OCT compound. The filled mold was placed in an insulated container with dry ice and solidified for 1 h. The solidified blocks were then equilibrated in a cryostat chamber at −21 °C for a minimum of 4 h. Once ready, the frozen blocks were trimmed and mounted onto the sample holder. Coronal sections, 45 µm thick, were cut using a microtome (CM1850; Leica Microsystems, Wetzlar, Germany). The free-floating sections were temporarily stored in ice-cold 0.1 M PBS until further processing.

Free-floating sections were processed in 1.5 mL tubes with gentle agitation. The following steps were performed: (1) antigen retrieval using 20 mM sodium citrate buffer (pH 9) for 2 h at 60 °C with gentle agitation; (2) pre-incubation in 1% sodium borohydride for 20 min at 37 °C; (3) incubation in a blocking solution consisting of 0.1 M PBS with 10% normal goat serum and 0.3% Triton X-100 for 2 h at RT; (4) incubation in the primary antibody, dissolved in an antibody solution composed of 0.1 M PBS with 1% BSA, 5% normal goat serum and 0.3% Triton X-100 for 24 h at 4 °C; (5) washing with 0.1 M PBS containing 0.3% Triton X-100 three times at RT with gentle shaking; (6) incubation in the secondary antibody, dissolved in antibody solution, for 2 h at RT; (7) washing with 0.1 M PBS containing 0.3% Triton X-100 three times at RT with gentle shaking; (8) mounting the sections onto slides in a 0.1 M PBS bath and immediately staining with mounting medium containing DAPI (Abcam, Cambridge, UK); (9) applying cover slips and sealing the edges with nail polish. The slides were left to dry overnight at 4 °C.

The primary antibodies used in this study included rabbit anti-glutamate 1 receptor (GluR1) (1:1000, Chemicon International, Temecula, CA, USA), rabbit anti-glutamate 2/3 receptor (GluR2/3) (1:1000, Chemicon International, Temecula, CA, USA), and mouse anti-glial fibrillary acidic protein (GFAP) (1:1000, Chemicon International, Temecula, CA, USA). To visualize the antibodies, polyclonal anti-rabbit Alexa Fluor^®^ 594 and monoclonal anti-mouse Alexa Fluor^®^ 488 (Molecular Probes, Inc., Oregon, USA) were used. Imaging was performed using a Zeiss Axioplan microscope (Carl Zeiss Meditec, Inc., Jena, Germany). Fluorescence images were captured using a 40× or 10× objective.

### 2.13. Statistical Analysis

Solubilized β-glucan samples were analyzed in triplicate and data were expressed as mean ± SEM. Statistical analysis was conducted using SPSS software v.20 (SPSS Inc., Chicago, IL, USA) at a 5% significance level. Samples (*n* = 5 per group) were analyzed in triplicate and data were expressed as mean ± SEM unless indicated otherwise. A student’s *t*-test was used to calculate the significance of differences compared to the Control group (*p* < 0.05).

## 3. Results

### 3.1. Optimization of Extraction and Solubilization Process

**Figure 2:**
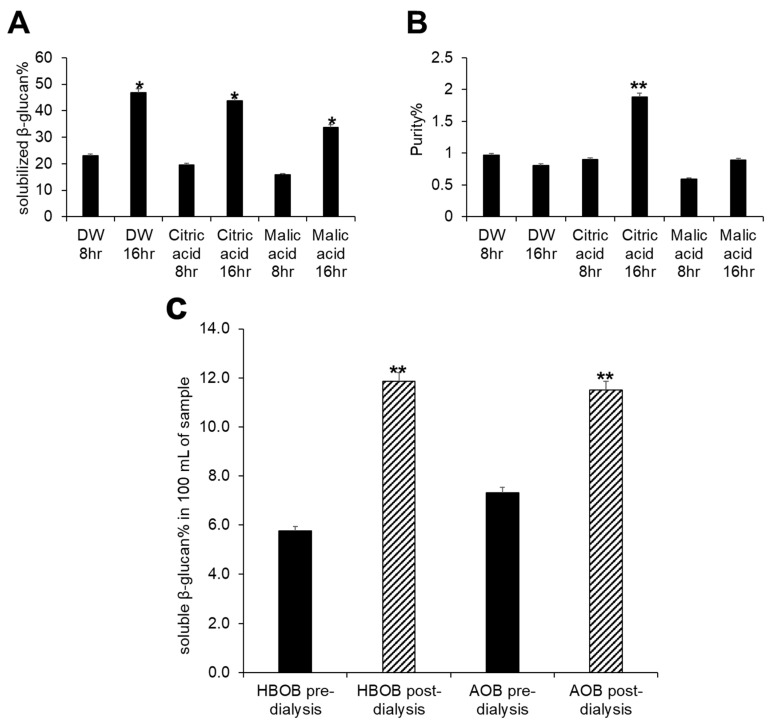
Analysis of β-glucan content in various samples using the mixed-linkage β-glucan assay kit. (**A**) Percentage of β-glucan solubilized via hydrolysis, based on the known β-glucan content of HBOB as specified by the manufacturer. (**B**) Purity of soluble β-glucan solutions under various hydrolysis conditions. (**C**) Purity of soluble β-glucan solutions before and after dialysis of β-glucans solubilized from HBOB and AOB via acid hydrolysis using 0.1 M citric acid. Significance was determined using a *t*-test to compare changes in purity or solubility over time or relative to the initial concentration, at 5% significance level as denoted by * or 1% significance level as denoted by **.

Acid hydrolysis using 0.1 M citric acid for 16 h at 85 °C produced a similar percentage of solubilized β-glucans as achieved with pure distilled water (Figure 2A). Notably, acid hydrolysis employing citric acid increased the purity of the resultant solution (Figure 2B). Therefore, acid hydrolysis with 0.1 M citric acid for 16 h at 85 °C was employed to solubilize β-glucan from HBOB and AOB. This was followed by digestion using pancreatin and dialysis to purify the solubilized oat β-glucan extract (Figure 1). Dialysis resulted in increased purity, as indicated by the doubling of soluble β-glucans post-dialysis (Figure 2C).

### 3.2. Yield of Solubilized β-Glucans

Acid hydrolysis using 0.1 M citric acid for 16 h at 85 °C resulted in approximately 9.76 g β-glucan per 100 g of dried extract (Table 2). Purification increased the β-glucan content in AOB to 66.98 g per 100 g of dried extract for each batch processed.

### 3.3. Molecular Weight of Solubilized β-Glucans

The weight-average molecular weight (Mw) of the solubilized β-glucan was 466.6 ± 0.80 kDa, which is consistent with values reported for oat and barley β-glucans solubilized using other methods [74,75]. The broad molecular weight distribution of solubilized oat β-glucan (polydispersity, Mw/Mn) was 2.71. Similarly, β-glucans from millet, sorghum, and barley solubilized using the same process, resulted in high-quality samples with lower yields (Supplementary Table S1). Monosaccharide composition analysis revealed that solubilized oat β-glucan consisted of 92.1% glucose and 7.89% galactose units (Supplementary Figure S1). The presence of galactose residues contributes to the heterogeneity of β-glucan and is positively correlated with branching degree, which is associated with high immunostimulatory activity [76].

### 3.4. β-Glucan Influences Weight Gain and Intestinal Barrier Function during Gestation

**Figure 3:**
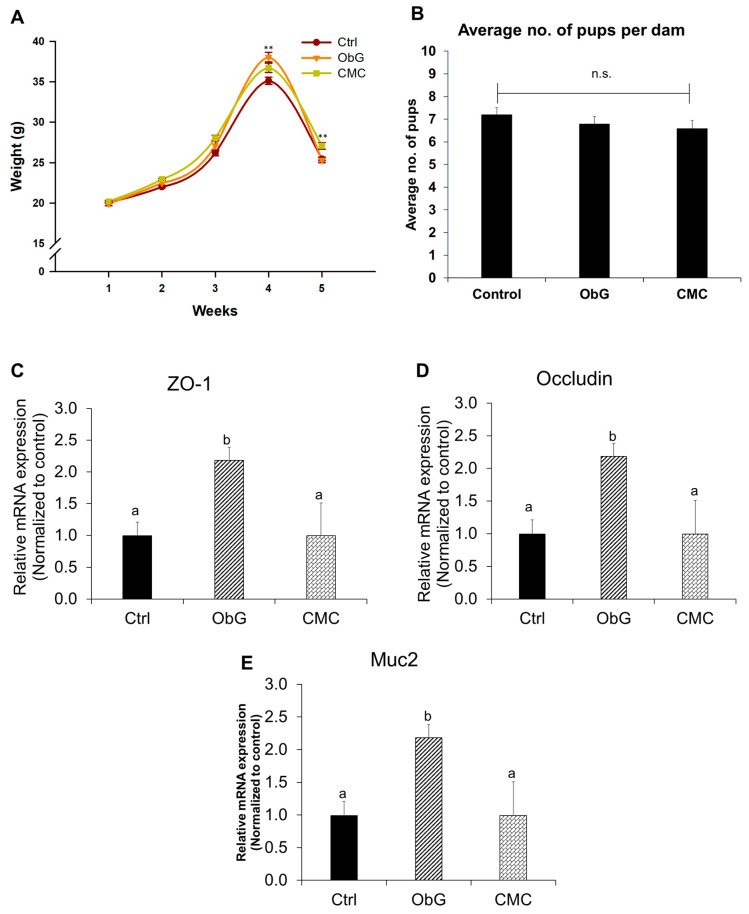
Clinical parameters observed in dams during gestation and birth (*n* = 6). (**A**) Trend in average body weight of dams 1 week before, during, and 1 week after gestation. (**B**) Average number of pups per dam in the three test groups. RT qPCR analysis of markers of intestinal barrier function: (**C**) ZO-1, (**D**) Occludin, and (**E**) Muc-2. Statistical significance was determined using ANOVA among the three test groups (*p* < 0.05). RT-qPCR data are presented as mean ± SEM, with different letters indicating significant differences in the ObG and CMC groups compared to the Control group, as determined using a *t*-test (*p* < 0.05).

The body weight of mice was recorded starting one week prior to gestation, continuing throughout gestation, and during the week following birth. Significant weight gain was observed during the final week of gestation in the ObG group, which exhibited greater weight gain compared to the Control and CMC groups (Figure 3A). However, one-week post-delivery, the CMC group had a significantly higher average weight than the Control group, whereas the ObG group showed an average weight comparable to the Control group. Notably, no significant difference was observed in the number of pups per dam across all groups (Figure 3B). This pattern of body weight gain has also been observed in pregnant rats fed 1.1 g per kg bw of oat β-glucan, which was associated with increased fetal body weight and length [77]. The weight gain during gestation, followed by a reduction post-delivery, may suggest increased fetal growth.

Tight-junction proteins and mucins are crucial components of the intestinal barrier, playing a vital role in maintaining homeostasis and the integrity of intestinal epithelial tissue [40]. SCFAs, such as butyrate, absorbed by gut epithelial cells, regulate cell differentiation, proliferation, and apoptosis, thereby enhancing the expression of tight-junction proteins, including Zonula Occludens-1 (ZO-1) and Occludin, and improving intestinal barrier function [78]. Dams in the ObG group exhibited significantly higher expression of these markers in the epithelial layer compared to the Control group, which had expression levels similar to the CMC group (Figure 3C,D). Strengthening the mucosal layer covering the intestinal epithelium helps prevent epithelial damage [40]. In this study, the expression of Mucin 2 (Muc2) increased in the ObG-supplemented dams compared to the Control group, which showed levels comparable to the CMC group (Figure 3E).

### 3.5. Cecal SCFA Analysis

**Figure 4:**
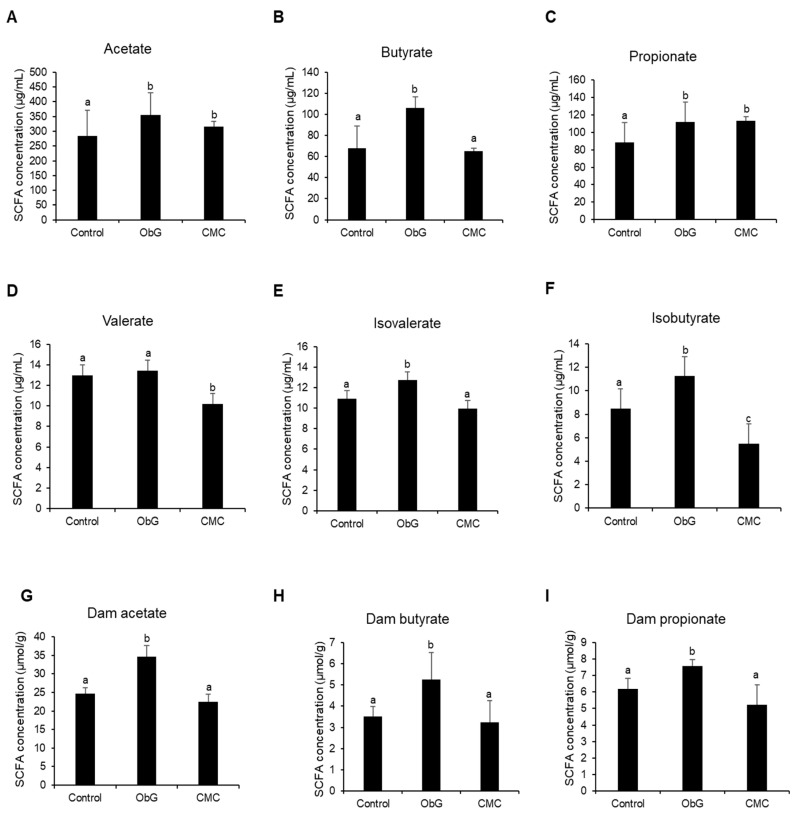
Alterations in cecal SCFA concentrations in 4-week-old pups (*n* = 6) (**A**–**F**) compared to dams (*n* = 4) (**G**–**I**) across various treatment groups. Data are presented as mean ± SEM. Different letters indicate significant differences between the ObG and CMC groups compared to the Control, as determined using a *t*-test (*p* < 0.05).

β-glucan serves as a fermentation substrate for gut microbes, such as lactobacilli, leading to the production of SCFAs [79]. In this study, cecal samples from 4-week-old pups in each group were analyzed for SCFA content. The ObG group exhibited the highest butyrate content (Figure 4B). The CMC and ObG groups showed higher levels of acetate (Figure 4A) and propionate (Figure 4C) compared to the Control group. Low-concentration SCFA iso-butyrate levels (Figure 4F) were significantly higher in the ObG group; however, valerate (Figure 4D) and iso-valerate levels (Figure 4E) did not exhibit significant differences across the three test groups. This trend was also observed in the cecal SCFA content of the dams (Figure 4G,H), with the exception of propionate levels (Figure 4I), which were reduced in dams in the CMC group. These findings suggest an increase in SCFA-producing bacteria in both dams and pups in the ObG-supplemented group. Such dietary changes are known to increase the abundance of beneficial bacterial families, including *Prevotellaceae*, *Lachnospiraceae*, and the *Bifidobacterium* genus [80].

### 3.6. Gut Microbiome Profile of Dams and 4-Week-Old Pups

**Figure 5:**
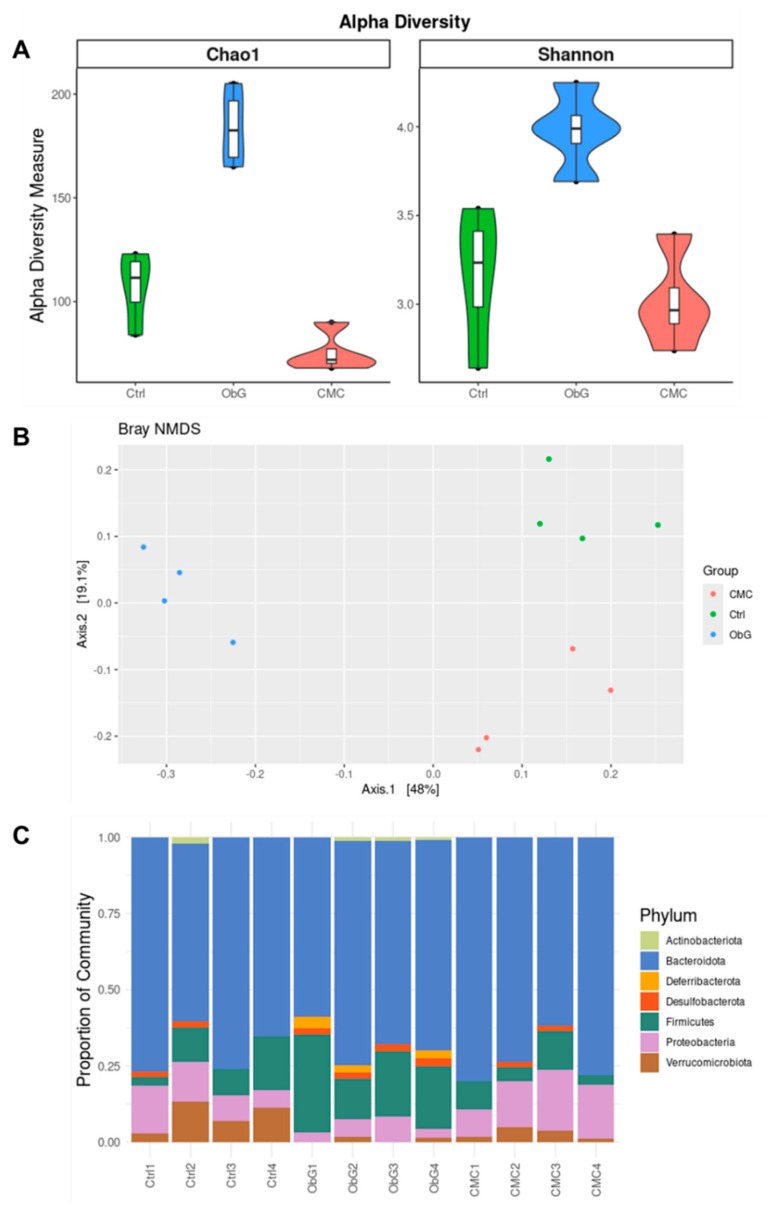
Effect of ObG supplementation on the gut microbiome profile in dams (*n* = 4). (**A**) Shannon diversity index of microbial alpha-diversity, based on relative abundance at the genus level, compared to the CMC and Control groups. (**B**) Comparison of relative abundance at the family level between the CMC and Control groups. (**C**) Principal coordinate analysis (PCoA) of genus profiling data from the three groups (ObG, CMC, Control), based on Bray-Curtis dissimilarity.

**Figure 6:**
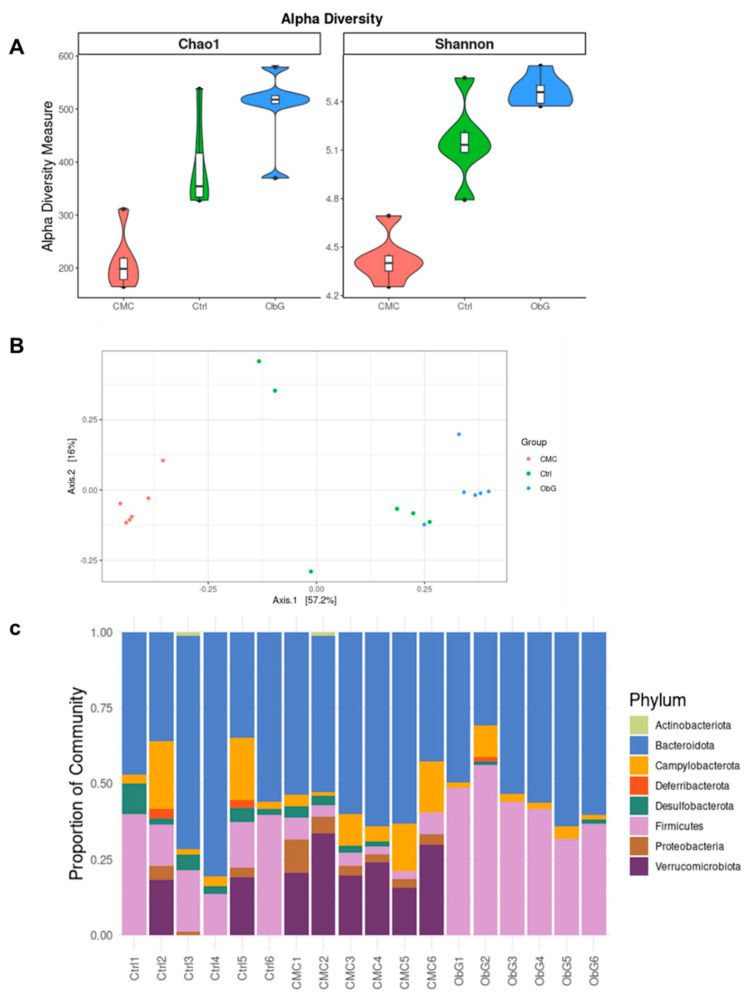
Effect of ObG supplementation on the gut microbiome profile of 4-week-old pups (*n* = 6). (**A**) Shannon diversity index of microbial alpha-diversity based on relative abundance at the genus level compared to the CMC and Control groups. (**B**) Comparison of relative abundance at the family level between the CMC and Control groups. (**C**) Principal coordinate analysis (PCoA) of genus-level profiling data in the three groups (ObG, CMC, Control), based on Bray-Curtis dissimilarity.

Previous studies have demonstrated that high species α-diversity, which refers to the diversity of microbiota within an individual, is associated with greater resistance to dysbiosis and a healthier microbiome [81]. Based on Chao1 and Shannon indices, α-diversity in dams from the ObG group was comparable to that of the Control group. In contrast, the CMC group exhibited significantly reduced α-diversity (Figure 5A). This indicates that ObG supplementation enhances resistance to dysbiosis during gestation. Similarly, in weaning pups (Figure 6A), α-diversity, as measured by both the Chao1 and Shannon indices, was higher in the ObG group compared to the Control group but significantly lower in the CMC group, indicating that increased soluble cellulose may negatively impact gut microbiome richness.

β-diversity, in contrast, describes the variation in microbial composition between individual sample sets or samples from different groups. It is computed by aggregating an individual’s microbial data into a single coordinate point and measuring the distance (using various metrics such as Bray-Curtis dissimilarity or unweighted and weighted UniFrac) between this point and that of another sample or group [82]. The differences between the groups were highlighted through PCoA based on Bray-Curtis dissimilarity (Figure 5B). This analysis revealed that dams in the ObG group had a distinct and uniform gut microbiome profile compared to the Control and CMC groups, which exhibited similar profiles. Notably, the gut microbiome profile of the weaning pups in the ObG group was not markedly different from that of the Control group (Figure 6A), likely due to the lack of uniformity in the microbiome profile of the Control group. Weaning pups in the CMC group displayed a significantly different profile compared to the Control and ObG groups (Figure 6A).

The Lachnospiraceae family, which belongs to the phylum Firmicutes, is a key component of the core gut microbiota and a major producer of SCFAs [83]. Additionally, this family has been shown to exert anti-inflammatory and immunomodulatory effects in chronic inflammatory diseases [84]. In this study, both dams and pups in the ObG group exhibited a significantly higher abundance of the Firmicutes phylum compared to the Control group, while the abundance was markedly reduced in the CMC group (Figure 5C and Figure 6C, respectively). Furthermore, the Lachnospiraceae family was more predominant in 4-week-old pups (Supplementary Figure S2). This finding aligns with the SCFA concentration analysis, which revealed that the ObG group had the highest overall SCFA production. Members of the Pseudomonadota phylum, formerly known as Proteobacteria [85], are known to produce pro-inflammatory molecules such as lipopolysaccharides, and their expansion has been linked to colitis and psychosocial stress in mouse models [86]. In the ObG-supplemented dams, the abundance of Pseudomonadota was lower compared to the Control group, whereas the CMC group showed a higher proportion in the overall microbiome profile (Figure 5C). Similarly, the gut microbiome profile of the 4-week-old pups exhibited an even lower abundance of Pseudomonadota (Figure 6C). A reduced abundance of the genus Akkermansia has been associated with higher systemic inflammation scores, including elevated levels of the pro-inflammatory cytokine TNF- α [87]. Pups in the CMC group exhibited a high abundance of the Akkermansiaceae family, whereas the ObG group showed reduced levels (Supplementary Figure S2). Notably, IL-10, which is upregulated with a reduced abundance of the Akkermansia genus [88], was downregulated in the ObG group. This suggests that another microbial taxon may have mitigated the potential negative effects associated with the reduced Akkermansia abundance. Members of the Muribaculaceae family, known to forage mucin monosaccharides in the intestinal tract, may contribute to the enhanced external “anatomical” barrier [89]. The ObG group exhibited high levels of the Muribaculaceae family, comparable to the Control group and significantly higher than the CMC group (Supplementary Figure S2).

### 3.7. Serum Cytokine Profile in Weaning Pups

**Figure 7:**
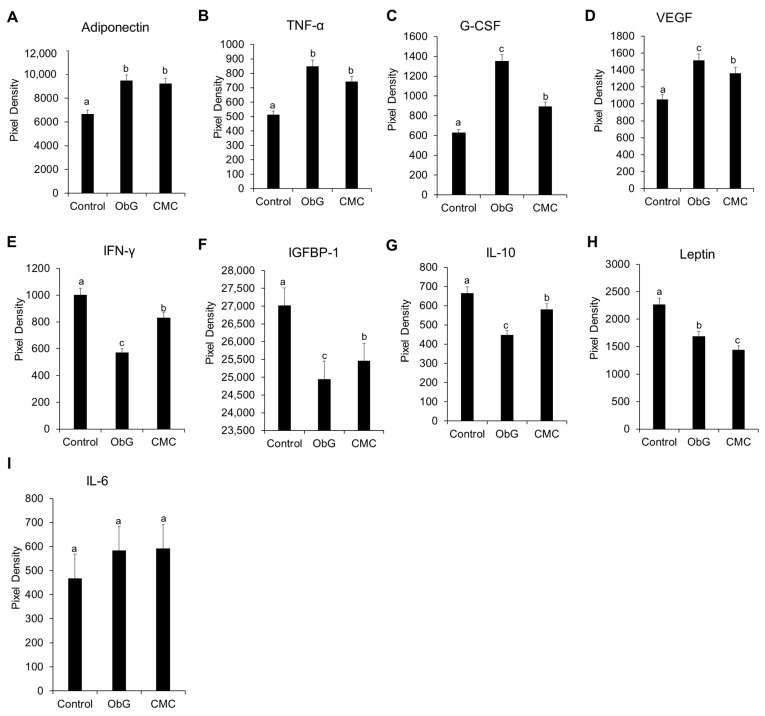
Alterations in serum cytokines levels of metabolic regulators (**A**–**I**) (Leptin, adiponectin, IGFBP-1), pro-inflammatory cytokines (TNF-α, IL-6, and VEGF), and anti-inflammatory cytokines (IL-10 and IFN-γ) following HOBG supplementation, compared to the CMC and Control groups (*n* = 8). Data are presented as mean ± SEM. Different letters denote significant differences in the ObG and CMC groups compared to the Control group, as determined using a *t*-test (*p* < 0.05).

Cytokine levels related to immunomodulation and metabolic regulation were analyzed in the serum of 4-week-old pups using a cytokine proteome profiler array. Both the ObG and CMC groups showed elevated levels of circulating adiponectin (Figure 7A) compared to the Control group. Adiponectin is a metabolic regulator known for its antioxidative and anti-inflammatory properties, often associated with the prevention of mitochondrial impairment [90]. Conversely, circulating levels of leptin (Figure 7H) were significantly reduced in the ObG group, with an even greater reduction observed in the CMC group compared to the Control group. Leptin stimulates the expression of pro-inflammatory cytokines in the placenta and various organs during the perinatal period [91]. Anti-inflammatory cytokines, including IL-10 (Figure 7G) and IFN-gamma (Figure 7E) [92,93], were most significantly decreased in the ObG group compared to the Control group. However, granulocyte colony-stimulating factor (G-CSF) (Figure 7C), which inhibits the allogenic immune response by modulating TNF-α secretion from monocytes/macrophages [94,95], was significantly high in the ObG group compared to both the Control and CMC groups. Insulin-like growth factor-binding proteins (IGFBP-1), which regulate glucose metabolism and insulin sensitivity by inhibiting insulin activity [96], were also assessed. Increased circulating IGFBP-1 levels in pups in the ObG group (Figure 7F) may indicate an upregulation of glucose metabolism and increased insulin sensitivity compared to the CMC and Control groups. In rodents, there is a positive correlation between the area of microglial populations in the cerebellum and basal ganglia, with proliferation occurring during the postnatal period and stable populations being established by day 28 [97]. The activation of microglia by pro-inflammatory cytokines such as TNF-α (Figure 7B) and VEGF (Figure 7D) in 4-week-old pups may promote microglial neurogenesis. Notably, IL-6 levels (Figure 7I) in the serum of all three test groups did not show significant differences, suggesting the absence of an inflammatory state. This lack of inflammation may have been influenced by increased SCFA production in the gut.

### 3.8. Comparison of Differentially Enriched Pathways in the Cerebral Cortex of 4-Week-Old Pups from the ObG and CMC Groups

**Figure 8:**
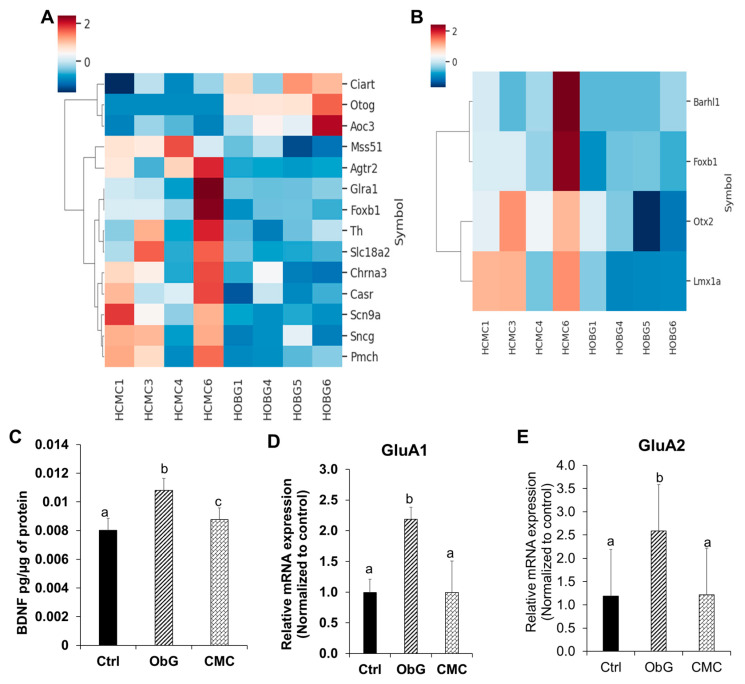

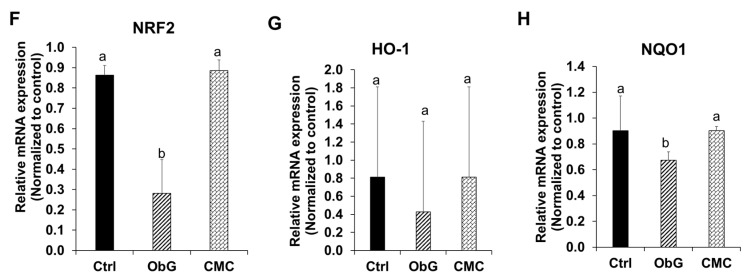
Differentially expressed genes in the cortex of 4-week-old pups from the ObG (labeled as HOBG) and CMC (labeled as HCMC) groups. (**A**) Top 14 differentially expressed genes. (**B**) Genes involved in the dopaminergic pathway. (**C**) mRNA markers for neurodevelopment in the brain, including BDNF levels in the brains of 1-week-old pups (*n* = 4) measured using the BDNF ELISA assay kit. (**D**) RT qPCR analysis of AMPA receptors GluA1, (**E**) GluA2, (**F**) Nrf2, (**G**) HO-1, and (**H**) NQO1. Data are presented as mean ± SEM. Differentially expressed genes shown have a log10-fold change > 2 and *p* < 0.05. Different letters denote significant differences in the ObG and CMC groups compared to the Control, as determined using a *t*-test (*p* < 0.05).

Differences in the pathways enriched by the supplementation of either ObG or CMC were investigated. Pathway enrichment analysis, utilizing the functional annotations of significantly differentially expressed genes, revealed several pathways that were over-represented in the ObG group compared to the CMC group (Supplementary Figure S3A,B). Notably, the dopaminergic neurogenesis pathway was significantly elevated in the ObG group compared to the CMC group (Supplementary Figure S3B). This effect is highlighted by changes in genes such as Otx2 and Lmx1a, which are known to be overexpressed in offspring of weaning age as a result of maternal immune activation [98]. The *Lmx1a* gene is a regulatory gene belonging to the LIM homeobox family, and is essential for neural development and differentiation in the midbrain [99]. A previous study found that overexpression of the LMXA1 protein in dopaminergic cell lines produced downstream effects on insulin receptor binding and calcium trafficking, potentially linking it to the later-life development of Parkinson’s disease [100,101]. Otx2, another homeobox gene, controls the regionalization of the vertebrate brain during embryonic development [102]. Its uptake by parvalbumin-positive cells during the postnatal period promotes their maturation and regulates the onset of critical periods in these cells. Critical periods of brain plasticity correspond to defined developmental stages during which experiences can shape learning and induce experience-based changes in neural circuitry [103]. A previous study reported that decreased expression of Otx2 in Otx2-het mice resulted in reduced anxiety-like behavior [104]. The ObG group displayed a significantly lower expression of *lmxa1* and *Otx2* RNA compared to the CMC group, where their expression levels were elevated (Figure 8B). This may indicate significant differences in the development of neuronal plasticity during this critical period. These observations are further supported by the increased expression of the *Casr* gene (Figure 8A), which is involved in calcium-sensing receptors, suggesting a potential alteration in calcium trafficking across cell membranes [105]. The *Pmch* gene, a precursor to the hypothalamic neuropeptide melanin-concentrating hormone, plays a critical role in the early development of normal energy homeostasis and has been shown to influence feeding motivation [106,107]. The reduced expression of the *Pmch* gene observed in the ObG group (Figure 8A) is associated with lower and stabilized body weight [108].

### 3.9. Perinatal Changes in mRNA Markers of Neurodevelopment and Memory

BDNF, a member of the neurotrophin family of growth factors, is ubiquitously expressed in the central nervous system and is critical for multiple neuronal processes, including neurogenesis, neuronal differentiation, long-term potentiation (LTP), learning, and memory [109,110,111,112]. In this study, high fiber intake was associated with increased BDNF levels in the brains of 1-week-old pups, with the ObG group showing the most significant increase compared to the Control group (Figure 8C). Alpha-amino-3-hydroxy-5-methyl-4-isoxazole propionic acid (AMPA) is an ionotropic glutamate receptor that functions through the flux of Na^+^ and Ca^++^ ions. It consists of various subunits, including GluA1 and GluA2, in a mature and excitatory state [113,114]. Learning and memory deficits associated with high-fat and high-sugar diets have been linked to decreased activity-dependent synaptic plasticity in the hippocampus and dysregulation of AMPA-type glutamate ionotropic receptors (AMPARs) that are crucial for neuroplasticity [113]. In this study, mRNA expression levels of GluA1 and GluA2 were significantly increased in 1-week-old pups from the ObG group (Figure 8D,E) compared to the Control group, whereas the CMC group showed no significant changes. The precise mechanism by which increased synaptic strength and LTP are mediated through AMPAR excitation, which suppresses NMDAR activity, remains under investigation [115,116,117].

Additionally, activation of Nrf2 by linalool has been shown to reverse the decreased expression of synaptic plasticity-related proteins, including calcium-calmodulin-dependent protein kinase II (CaMKII), p-CaMKII, and BDNF [117]. Consistent with this, Nrf2 expression in the brains of 1-week-old pups was significantly reduced compared to the Control group, whereas the CMC group showed expression levels similar to those of the Control group (Figure 8F). Interestingly, the expression of HO-1 within the Nrf2/HO-1 antioxidant stress pathway was not significantly altered among the three groups. In contrast, NQO1, a multifunctional antioxidant regulated downstream of Nrf2 activation, was suppressed in the ObG group (Figure 8G,H). These findings suggest that incorporating ObG into the diet of dams may enhance neurodevelopment and synaptic plasticity in 1-week-old pups.

### 3.10. Perinatal Alterations in Protein Markers of Neurodevelopment and Memory

**Figure 9:**
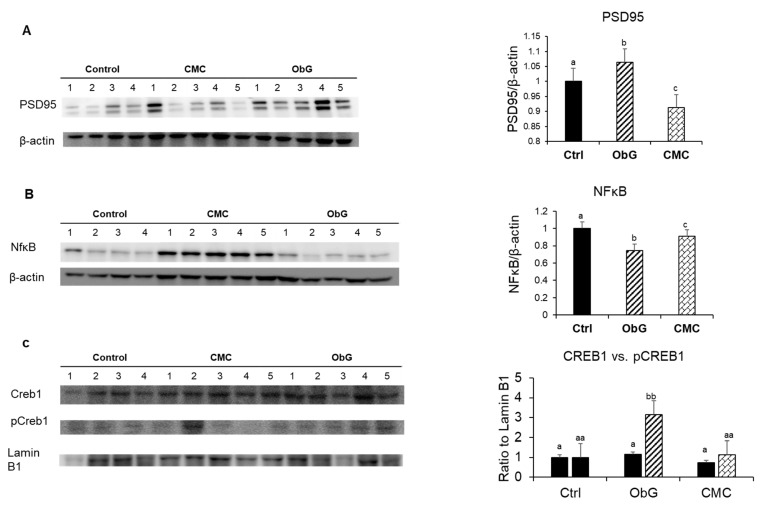
Protein markers associated with neurodevelopment in the brains of 1-week-old pups using western blots (*n* = 5). Levels of (**A**) PSD95, (**B**) NFκB, (**C**) CREB-1, and pCREB-1, with β-actin and Lamin B1 serving as controls. Data are presented as mean ± SEM. Different letters denote significant differences in the ObG and CMC groups compared to the Control group, as determined using a *t*-test (*p* < 0.05).

BDNF is influenced by key transcriptional regulators in neurons, including CREB and NF-κB, whose activation results in increased expression of BDNF [112,118,119,120,121]. Consistent with the observed increase in BDNF mRNA expression, levels of pCREB (activated CREB) were also elevated in the brains of 1-week-old pups from the ObG group compared to the Control group, whereas CMC supplementation showed no significant change (Figure 9C). Notably, NF-κB levels were significantly decreased in the ObG group, suggesting that this pathway may not be associated with the observed increases in BDNF and PSD95 (Figure 9A and Figure 9B, respectively). A previous study investigating the effects of subarachnoid hemorrhage found that neuroinflammation is driven by the generation of reactive oxygen/nitrogen species (ROS/RNS), which perpetuate neuronal degeneration through oxidative stress mediated by the NF-κB pathway [122]. This phenomenon is also characteristic of stress-induced neuropsychiatric disorders, such as depression and cognitive impairment [123]. The reduced expression of the antioxidant-related gene NQO1 and the under-expression of NF-κB in the ObG group suggest that supplementation may have mitigated oxidative stress and neuroinflammation. PSD95 indirectly binds to AMPAR and regulates synaptic strength, primarily through its overexpression, which stabilizes young synaptic contacts, though it does not affect older neurons [124]. In 1-week-old pups, PSD95 levels were increased in the ObG group, suggesting a potential enhancement in synaptic strength during the perinatal stage compared to the Control group (Figure 9A).

### 3.11. Solubilized Oat β-Glucan Increases the Expression of Neurodevelopmental and Synaptic Strength Markers in the Hippocampus

**Figure 10:**
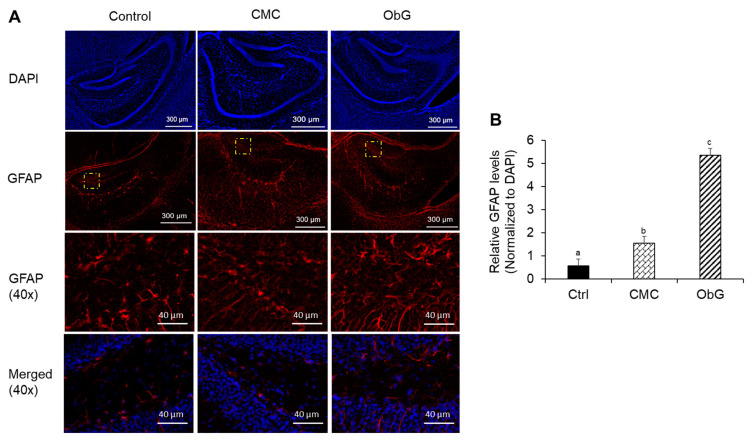
Immunohistochemistry of neurodevelopmental markers in the hippocampus of 4-week-old pups in the Control, CMC, and ObG test groups. (**A**) Representative images of hippocampus sections stained with GFAP and DAPI at 10× and 40× magnification. The section demarcated by the yellow box in the second row of panels shows the area that was further magnified to 40× (**B**) Relative GFAP protein levels represented as fluorescence intensity normalized to DAPI in 40× magnified images of the dentate gyrus region of the hippocampus. Different letters denote significant differences in the ObG and CMC groups compared to the Control group, as determined using a *t*-test (*p* < 0.05).

Most neuronal connections in the brain are established during development and are continually remodeled throughout life in response to various conditions [125]. In the mouse cortex and throughout the forebrain, nearly all astrocytes are derived from GFAP-expressing progenitor cells [126]. These immature astrocytes undergo morphological and functional maturation during the first few weeks of postnatal development, establishing intimate relationships with synapses, as seen in the dentate gyrus [127,128]. The expression of GFAP, a marker for mature and reactive astrocytes, in the hippocampus (Figure 10A) and the observed increased branching was significantly higher in the ObG group compared to the Control and CMC groups [129]. These findings indicate increased synaptic formation and strength. The reduction in AMPAR and NMDAR subunits results in a reduction in LTP amplitude [130]. Consistent with the results obtained from the analysis of the brains of 1-week-old pups, the expression levels of GluR1 and GluR2/3 were marginally elevated in the brains of 4-week-old pups from the ObG group (Supplementary Figure S4).

### 3.12. Solubilized Oat β-Glucan Improves Learning, Long-Term Memory, and Cognition

**Figure 11:**
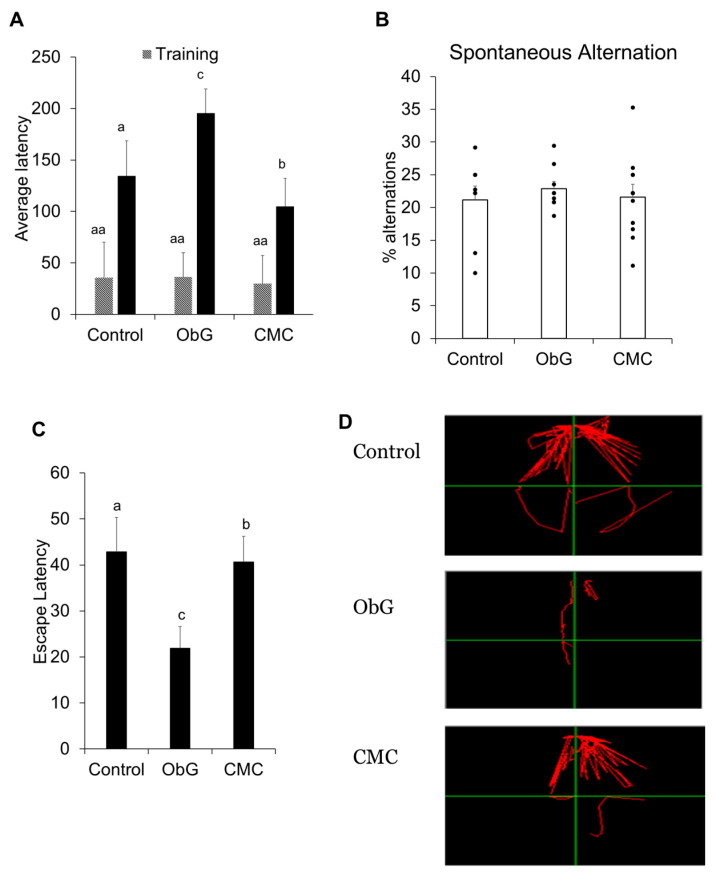
Cognition, learning, and long-term memory tests on 8-week to 11-week-old pups. (**A**) Average latency of mice in the light chamber during the passive avoidance test, both before and after training, across the three test groups. (**B**) Percentage alternations made by mice during the Y-maze test for spontaneous alternations across the three test groups. (**C**) Average escape latency of mice following training in the Morris water maze test. (**D**) Tracking mice movement of individual subjects in each of the three test groups. Data are presented as mean ± SEM. Different letters denote significant differences in the ObG and CMC groups compared to the Control group, as determined using a *t*-test (*p* < 0.05).

The passive avoidance task is a fear-aggravated test used to evaluate associative learning and memory in rodent models of CNS disorders. In this test, subjects learn to avoid an environment where an aversive stimulus, such as a foot shock, was previously delivered. The Morris water maze (MWM) is designed to test spatial learning and memory by measuring escape latency in the MWM water tank. Subjects were tracked using a video tracking system positioned directly above the water tank during swimming, and parameters were measured using tracking software on a computer. The ObG group exhibited the highest levels of associative and spatial learning and memory abilities, as indicated by increased latency in the passive avoidance test (Figure 11A) and shorter escape times in the Morris water maze test (Figure 11C,D) compared to the Control group. Conversely, the CMC group showed reduced cognitive abilities compared to the ObG group. Notably, the Control group exhibited higher cognitive function than the CMC group, indicating that high amounts of CMC in the diet may be detrimental to neurodevelopment (Figure 11A,C,D).

The Y-maze spontaneous alternation test measures the rodents’ willingness to explore new environments and assesses their working memory. Throughout multiple arm entries, subjects should exhibit a preference for entering less recently visited arms. The number of arm entries and the number of triads were recorded to calculate the percentage of alternation. No significant differences were observed among the three test groups (Figure 11B).

These results demonstrate significant differences in cognitive function between the different diets, suggesting distinct mechanisms by which CMC and ObG influence neurodevelopment. Furthermore, the enhanced neurodevelopment and synaptic strength observed during the perinatal stage may persist into adulthood, as evidenced by the ObG group exhibiting superior cognitive, learning, and memory capabilities.

## 4. Discussion

At concentrations exceeding 1%, solubilized β-glucan molecules organize into a viscous, pseudoplastic complex [36]. The optimized solubilization protocol in this study yielded viscous solutions containing β-glucans with low molecular weights ranging from 40 to 470 kDa. Extraction using hydrochloric or sulfuric acid at temperatures between 50 °C and 60 °C resulted in less degradation compared to alkaline extraction methods and was also cost-effective [131]. Similarly, the polydispersity index (Mw/Mn) from different oat cultivars in this study ranged from 1.50 to 2.39, comparable to the value of 2.71 found in this study. This suggests that lower molarity acids, such as citric acid, are safer and more economical alternatives to hydrochloric acid, without compromising the quality of the final product. The solubilization of oat β-glucan resulted in a product with the highest purity (66.98 ± 2.9 g β-glucan per 100 g of dried sample) and an average molecular weight of 466.6 ± 0.80 kDa. One of the primary challenges encountered was the time required for the protocol. Future studies should consider incorporating ultrasound or microwave extraction as an additional step or in conjunction with citric acid to expedite the extraction process.

Balanced maternal nutrition during pregnancy and lactation, along with the promotion of a healthy gut microbiota in both mother and child, may confer long-term health benefits [132]. Recent studies have shown that SCFAs play a role in regulating weight gain during pregnancy by improving glucose metabolism and influencing serum levels of various metabolic hormones [133]. For example, higher levels of acetic acid have been associated with both weight gain and increased levels of the metabolic regulator adiponectin, while higher propionic acid levels have been linked to lower leptin levels, a hormone that regulates satiety [134]. Among the dams, the average body weight increase during the final week of gestation was greater in the ObG group compared to the Control and CMC groups. Furthermore, both the dams and offspring in the ObG group exhibited significantly higher concentrations of cecal butyrate and acetate than those in the Control and CMC groups, with the dams also showing a higher proportion of propionate. Studies have demonstrated that SCFAs have the potential to counteract the dysfunctional energy metabolism and reduced insulin and leptin sensitivity often observed during pregnancy [135,136,137]. In one study involving both normal and overweight women, supplementation with high amounts of soluble commercial oat β-glucan resulted in a significant reduction in glucose and insulin responses [138]. These effects, as observed in this study, can be transmitted to offspring via the gut microbiota. In this study, cytokines involved in regulating lipid and glucose metabolism, such as adiponectin, IGFBP-1, and leptin, showed significant upregulation in the ObG offspring.

Additionally, the gut bacteria of vaginally delivered newborns closely resemble their mother’s vaginal microbiome [139]. This microbial fingerprint, established regardless of the exact timing of colonization, is dominated by beneficial bacteria such as *Lactobacillus*, *Bifidobacterium*, and *Prevotella* [140]. This microbiota plays a crucial role in enhancing gut barrier function and the development of the gut immune system [141]. The mucosal layer is a key component of the intestinal barrier, functioning to defend the body against the infiltration of toxic macromolecules and potentially harmful antigens and pathogens from the gut lumen [142]. Furthermore, the colonization and adherence of *Lactobacilli* to the intestinal mucosa inhibit pathogen attachment [143]. In this study, the 4-week-old ObG offspring exhibited increased expression of the intestinal epithelial muc-2 gene, which is responsible for mucin production [144]. The reduction in intestinal permeability is further evidenced by the increased expression of tight junction proteins, such as ZO-1 and occludins, in the ObG offspring. *Akkermansia muciniphila* has been implicated in several studies as contributing to the production of tight junction proteins and mucin [145,146]. Interestingly, the pups supplemented with CMC exhibited significant levels of *A. muciniphila*, whereas the ObG-supplemented dams showed increased levels of another family of mucin degraders, *Muribaculaceae*. The *Muribaculaceae* family, a dominant member of the *Bacteroidetes* phylum, possesses a strong capacity to metabolize both endogenous and exogenous polysaccharides, such as host glycans (mucins) and plant glycans [147]. This family has been reported to have a positive correlation with the growth of SCFA-producing bacteria such as *Lactobacilli* and *Bifidobacterium*, suggesting a possible cross-feeding relationship [148,149,150]. In this study, all three supplementation groups exhibited a significant proportion of the *Bacteroidetes* phylum. Notably, the ObG group had a more pronounced population of the *Firmicutes* phylum when compared to the Control and CMC groups. The inner “functional” immunological intestinal barrier is influenced by the *Firmicutes*/*Bacteroidetes* ratio, as these groups are the primary fermenters of dietary fiber, which in turn produces SCFAs [13,14,151].

In one study involving the transplantation of human maternal microbiota to germ-free mice, serum and stool levels of pro-inflammatory cytokines, such as IL-6 and TNF-α, were significantly elevated in the third trimester compared to the first, suggesting a potential link to gut inflammatory status [135]. It is well-established that maternal diet has a profound impact on the immunological development of offspring, as evidenced by changes in serological cytokines [152,153]. In this study, anti-inflammatory serum cytokines IL-10 and IFN-γ in 4-week-old pups were significantly reduced due to ObG supplementation. This finding may indicate the presence of a pro-active immunological intestinal barrier in the gut, characterized by a lower abundance of inflammation-inducing bacteria and their metabolites [154]. The decreased presence of members of the *Pseudomonadota* phylum, known to produce pro-inflammatory molecules such as lipopolysaccharides [86], in both the ObG dams and pups, underscores the changes in maternal gut microbiota and the potential transfer of this profile to their offspring.

Despite neonates having minimal gut microbiota on day 1, maternal factors, such as growth factors, milk polysaccharides, and microbiome metabolites such as SCFAs, play a crucial role in modulating responses to microbes during early life and are transmitted through milk [21]. A study by Nakajima et al. demonstrated that maternal dietary fiber intake during pregnancy and lactation influences the differentiation of intestinal Foxp3^+^ tTreg cells in the offspring mediated by circulating SCFAs, including acetate, propionate, and butyrate, derived from maternal milk [155]. This study found significant similarities in the microbiome profiles of dams and offspring in terms of α- and β-diversity, extending to the genera present in the gut of each group and the SCFAs produced by these probiotic bacteria. The findings of this study indicate that supplementation with solubilized AOB during gestation and early life has a significant impact on neurodevelopment, as evidenced by increased expression levels of PSD95 and BDNF in 1-week-old pups. Notably, soluble CMC did not produce the same effect, which may be attributed to differences in the linkage between cellulose and β-glucan. This variation could also be related to the solubilization method, as cellulose was modified through carboxymethylation, whereas oat β-glucan was hydrolyzed using citric acid to reduce its molecular weight. Behavioral changes observed in the upregulated pathways from RNA sequencing in 4-week-old pups, along with the differences noted in the fear conditioning test of 11-week-old pups in the ObG group, suggest that the neurodevelopmental effects are both extensive and long-lasting. The precise mechanisms by which neurogenesis is upregulated require further investigation, as various pathways were differentially influenced by changes in AMPA receptors, NF-κB, and phosphorylated CREB expression. The increased astrocyte development and reactivity, evidenced by pronounced GFAP expression in the stained hippocampus of 4-week-old pups in the ObG group, may have contributed to the enhanced spatial memory and learning abilities observed in these animals [129].

## 5. Conclusions

Solubilized oat β-glucan significantly improved gut health by promoting the growth of short-chain fatty acid-producing commensal and probiotic bacteria during pregnancy. This change is a key factor underlying the metabolic regulation, anti-inflammatory effects, and neurodevelopmental enhancement observed in the offspring. The transfer of gut microbiota at birth, followed by the establishment of core gut microbes during breastfeeding, are crucial events in the early life of the offspring. During this period, gut–brain axis crosstalk plays a critical role in growth and neurodevelopment as demonstrated in this study. The synaptic strength and long-term potentiation developed during this time significantly influence cognition and memory in adulthood. Inflammatory markers, regulated and produced in the gut through SCFA production serve as important messengers and drivers of neurogenesis and neurodevelopment. Given the increased bioactivity of soluble oat β-glucan compared to its insoluble form, it would be beneficial to expand the dietary intake recommendations for β-glucan to include soluble oat β-glucan, along with guidelines on solubilization processes and modifications to ensure maximum efficacy.

## Figures and Tables

**Table 1:** Primer sequences used for qPCR.

Gene	Forward (5′-3′)	Revere (5′-3′)	Tm Forward (°C)	Tm Reverse (°C)
GluA-1	AAGAGAAACAGAGAACCT	GATGTACGGCATATTCCTT	50.9	51.2
GluA-2	TTTGTCCATGCTCTACTT	ATCTGTATGGTGTTAGAAGA	49.5	50.0
HO-1	CAAGCAGAACCCAGTCTATG	GCGTGCAAGGGATGATT	54.9	54.2
Nqo1	GACAACGGTCCTTTCCAGAAT	CTCTGAATCGGCCAGAGAATG	57.1	57.6
Nrf2	TTCCTCTGTCCTTTCCAGAAT	GCTCTTCCATTTCCGAGTCAC	61.4	60.5
Gapdh	TCACCACCACCATGGAGAAGGC	GCTAAGCAGTTGGTGGTGCA	58.3	60.2
Muc2	CCTTAGCCAAGGGCTCGGAA	GGCCCGAGAGTAGACCTTGG	60.9	60.7
Occludin	ATGTCCGGCGATGCTCTC	TTTGGCTGCTCTTGGGTCTGT	61.2	61.1
ZO-1	ACAGGCCATTACGAGCCTCT	GGAGGCTGTGGTTTGGTAGC		
*β*-actin	GGCTGTATTCCCCTCCATCG	CCAGTTGGTAACAATGCCATGT	59.0	58.5

**Table 2:** The percentage of β-glucan in raw oat bran powder versus solubilized β-glucan extract from various cereals compared to a standard.

Sample	Purity (Grams of β-Glucan/100 g Dried Sample) ^1^
Megazyme™ standard Oat flour	6.71 ± 2.9
Solubilized Oat β-glucan	5.51 ± 2.5
Solubilized Barley β-glucan	3.56 ± 2.5
Solubilized Sorghum β-glucan	0.81 ± 2.7
Solubilized Millet β-glucan	0.63 ± 2.7
Solubilized AOB β-glucan extract powder	9.76 ± 2.3
Purified AOB β-glucan extract powder	66.98 ± 2.9

^1^ Values represent mean ± standard deviation (*n* = 3).

## Data Availability

The original contributions presented in the study are included in the article/Supplementary Materials, further inquiries can be directed to the corresponding author.

## Supplementary Materials

The following supporting information can be downloaded at: https://www.mdpi.com/article/10.3390/foods13193102/s1. Table S1. Purity of solubilized cereal β-glucans. Figure S1. HPLC spectrum showing monosaccharide profile of solubilized oat bran β-glucan. Figure S2. Cumulative bar graph of relative abundance at family level of 4-week old pups in of ObG (labeled as HOBG) and CMC (labeled as HCMC) groups and Control (labeled as LCMC). Figure S3. Analysis of enriched pathways using (A) Gene ontology and (B) Wikipathways platforms. Genes shown have a log10 fold change > 2 and *p*-value < 0.05. Figure S4. Immunohistochemistry of neurodevelopmental markers in the hippocampus of 4-week-old pups in Control, CMC, and ObG test groups. Representative images of hippocampus sections stained with (A) GluR1, and (B) GluR2/3 and DAPI, scale bar = 300 µm.
