# Supplementary material for: Solubilized β-Glucan Supplementation in C57BL/6J Mice Dams Augments Neurodevelopment and Cognition in the Offspring Driven by Gut Microbiome Remodeling
Source: Foods. 2024 Sep 28;13(19):3102. doi: 10.3390/foods13193102 (PMC11476385; doi:10.3390/foods13193102)
Supplement: Supplementary file 1 [file foods-13-03102-s001.zip › foods-3224732-supplementary.pdf]

**Supplementary Table S1.** Purity of solubilized cereal β-glucans

| Sample | Mw (×10³ gmol⁻¹)[1] | Mw/Mn[2] |
|---|---|---|
| Barley | 83.1 ± 0.90 | 2.79 |
| Sorghum | 41.1 ± 0.90 | 1.71 |
| Millet | 129.7 ± 0.95 | 3.17 |
| Oat Bran | 466.6 ± 0.80 | 2.71 |

[1]Weight-average molecular weight (Mw)

[2]Polydispersity (weight-average molecular weight (Mw)/number-average molecular weight (Mn))

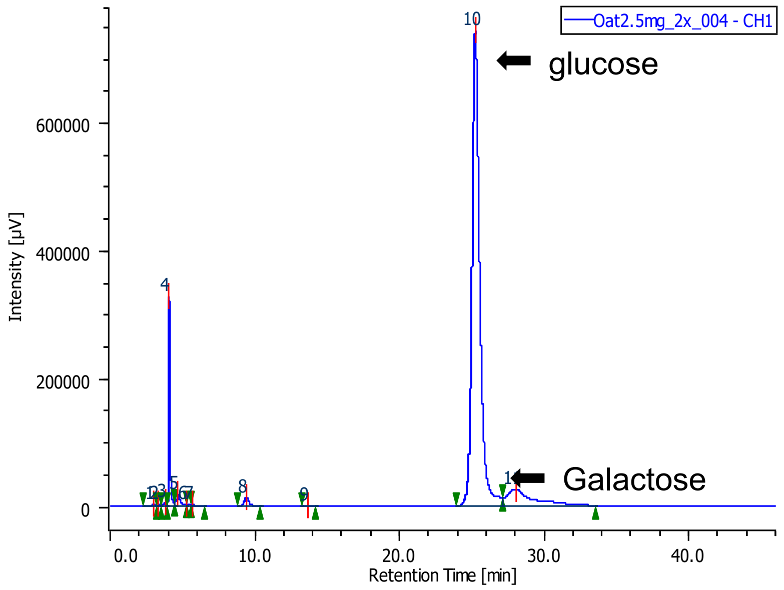


**Supplementary Figure S1:** HPLC spectrum showing monosaccharide profile of solubilized oat bran β-glucan

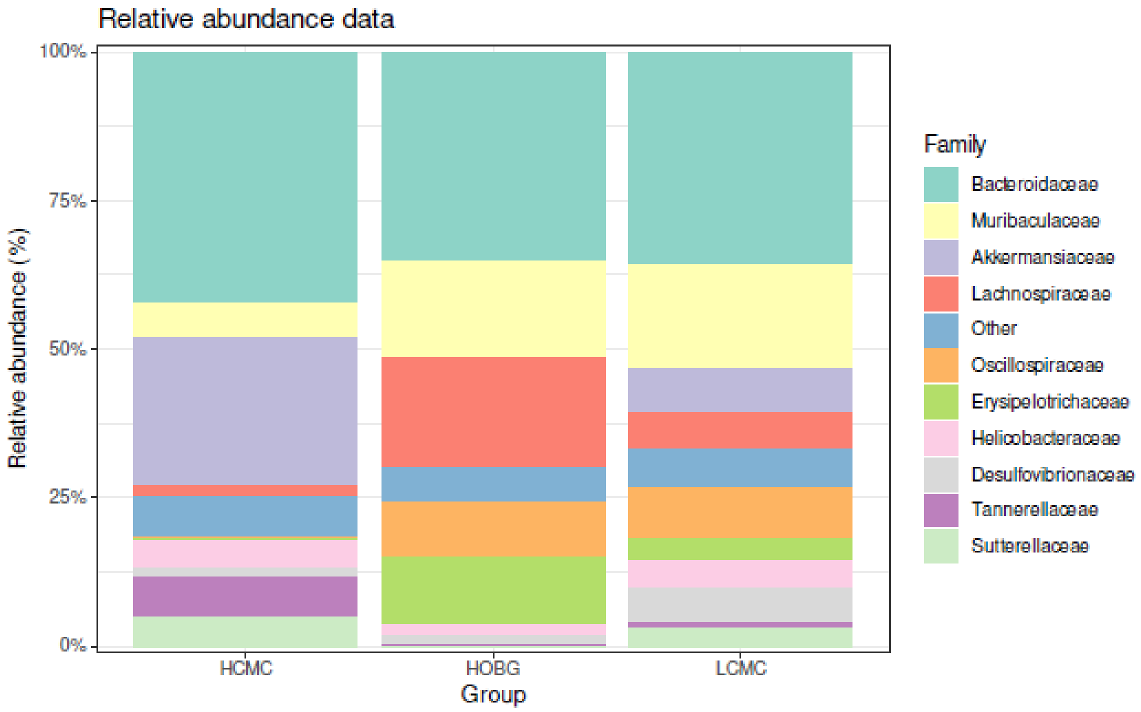


**Supplementary Figure S2**. Cumulative bar graph of relative abundance at family level of 4-week old pups in of ObG (labeled as HOBG) and CMC (labeled as HCMC) groups and Control (labeled as LCMC)

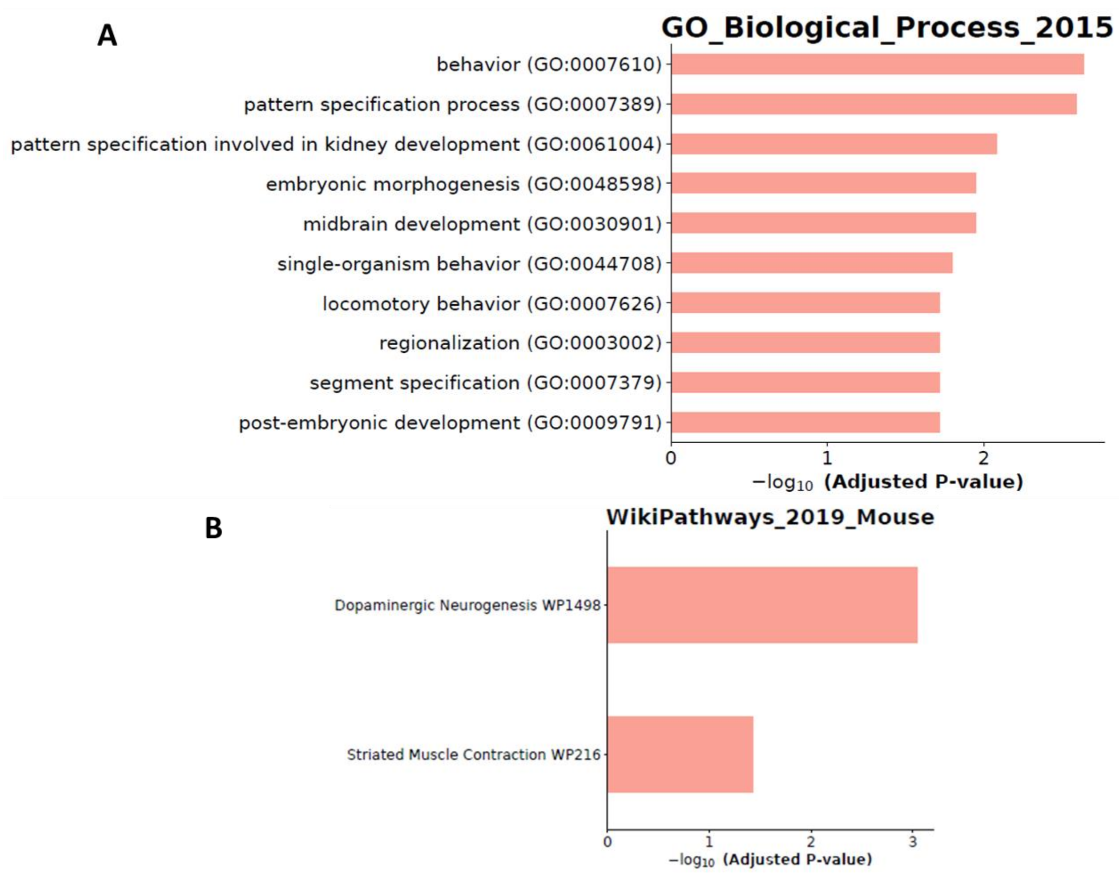


**Supplementary Figure S3.** Analysis of enriched pathways using (**A**) Gene ontology and (**B**) Wikipathways platforms. Genes shown have a log10 fold change > 2 and *p-value* < 0.05.

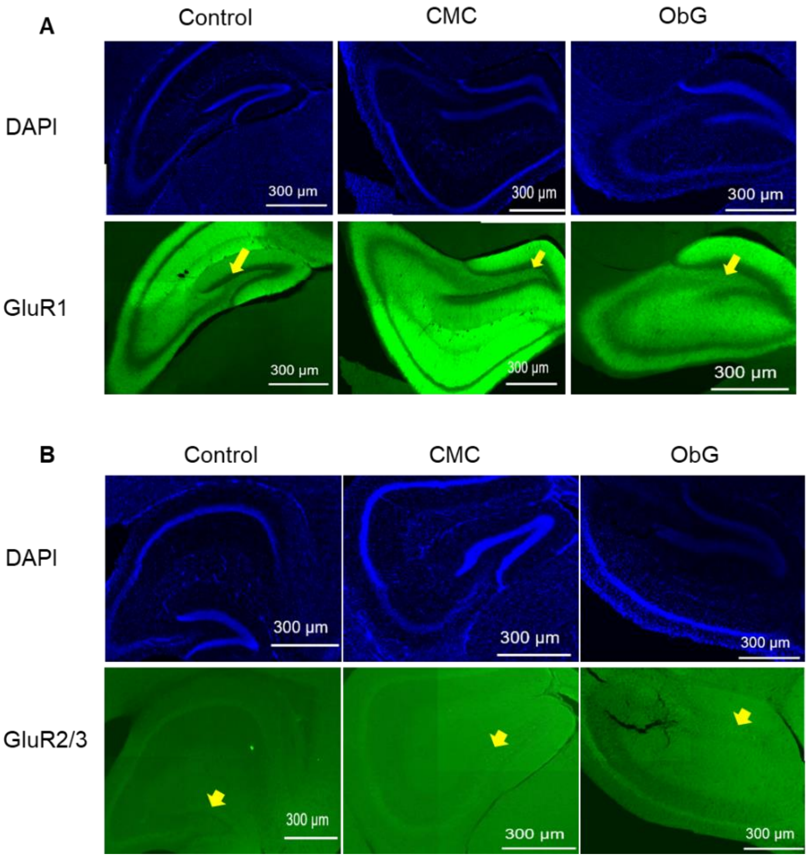


**Supplementary Figure S4**. Immunohistochemistry of neurodevelopmental markers in the hippocampus of 4-week-old pups in Control, CMC, and ObG test groups. Representative images of hippocampus sections stained with (**A**) GluR1 and (**B**) GluR2/3 and DAPI, scale bar = 300 µm.
